# Systematic Review and Meta-Analysis of Health-Related Quality of Life in Patients with β-Thalassemia that Underwent Hematopoietic Stem Cell Transplantation

**DOI:** 10.2174/17450179-v17-e211208-2021-HT2-1910-4

**Published:** 2023-12-10

**Authors:** Olga Mulas, Ilaria Pili, Marco Sanna, Giorgio La Nasa

**Affiliations:** 1 Ematologia e CTMO, Ospedale Businco, ARNAS “G. Brotzu”, Cagliari, Italy

**Keywords:** β-Thalassemia major, Allogenic hematopoietic stem cell transplantation, HRQoL, Transplant, Immunogenetic, Iron chelation therapy

## Abstract

**Background:**

β-Thalassemia major (β-TM) represents one of the most important hemoglobinopathies worldwide. Remarkable improvements have been achieved in supportive therapy based on blood transfusions and iron chelation, and nowadays, this approach is capable of assuring a long life in these patients in industrialized countries. The only curative treatment is represented by hematopoietic stem cell transplantation (HSCT). However, this treatment may be burdened by deterioration in the Health-Related Quality of Life (HRQoL). This paper aimed to evaluate the role of HRQoL in transplanted β-TM patients with a systematic review and meta-analysis.

**Methods:**

PubMed database, Web of Science, and Scopus were systematically searched for studies published between January 1st, 2000 to September 2020. The following terms were entered in the database queries: β-thalassemia, HRQoL, and HSCT. The study was carried out according to the Preferred Reporting Items for Systematic and Meta-analyses (PRISMA) statement.

**Results:**

We identified a total of 33 potential studies. Among these, 10 were finally considered in the systematic review and 5 in the meta-analysis. Overall, good scores in the principal domains of HRQoL were reported by transplanted patients. These data were confirmed by results of meta-analysis that showed significant difference between transplanted and β-TM patients treated with conventional therapy in the physical and emotional dimension, with a medium effect size [d=0.65, 95% CI (0.29-1.02), z = 3.52, p =0.0004, I^2^=75%; and d=0.59, 95% CI (0.43-0.76), z = 6.99, p <0.00001, I^2^=0%, respectively].

**Conclusion:**

HRQoL is generally good in β-TM transplanted patients and may significantly contribute in deciding whether or not to transplant a β-TM patient treated with conventional therapy.

## 1 BACKGROUND

Beta-thalassemia major (β-TM) is among the most common genetic diseases that affect millions of children throughout the world [1]. Over time, β-TM has been the protagonist of countless advances both in terms of understanding the molecular and pathogenic mechanisms that determine it, as well as in its treatment. Indeed, patients born in the last decades can have a life expectancy comparable to healthy individuals with standard therapy based on transfusion support and iron-chelation [2]. However, many differences remain between industrialized and developing countries, where the availability of economic resources for public health is still insufficient. The maintenance of a high standard of treatment requires a massive expenditure of resources that heavily affect health spending. This means that, especially in developing countries, where the disease occurs at a very high frequency, an adequate level of care cannot always be offered to the patients [3]. Hematopoietic stem cell transplantation (HSCT) is currently the only definitive cure for β-TM. It is the only one capable of determining definitive healing in a large number of patients, especially in those children and young adults who arrive at the transplant procedure regularly transfused and those who underwent a good iron-chelation regime [4]. Unfortunately, HSCT procedure can be burdened by short-term side effects (rejection, acute graft *versus* host disease - GVHD) and long-term side effects (chronic GVHD, major organ dysfunction, increased incidence of solid tumors), without a negligible risk of mortality [5-7]. A good outcome of HSCT is also related to the immunogenetic status of donors and recipients [8]. The final therapeutic choice is difficult and requires a careful evaluation of several aspects.

Health-related quality of life (HRQoL) is generally conceptualized as a multidimensional construct referring to patients’ perception of the impact of disease and treatment on their physical, psychological, and social functioning and well-being [9]. However, studies that have evaluated this aspect in the transplanted β-TM are very limited.

The aim of this paper was to perform a systematic review and meta-analysis of the main studies that have addressed the HRQoL in β-TM patients who underwent HSCT in comparison with conventional therapies, other diseases, or HRQoL of the general population, in order to assess HRQoL benefits of the transplant procedure.

## 2 MATERIALS AND METHODS

### 2.1 Search Strategy

**Fig. (1):**
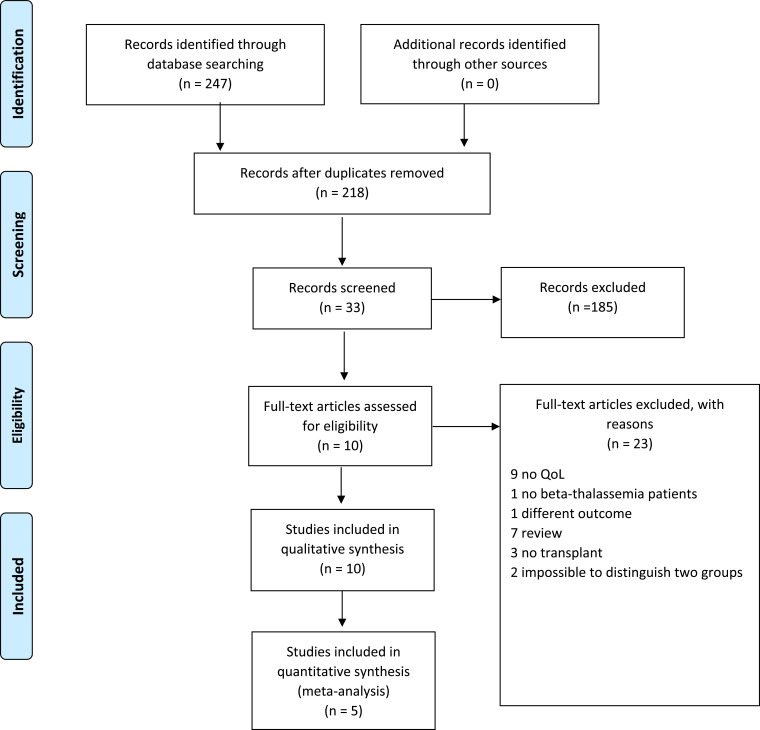
PRISMA 2009 Flow Diagram.

A systematic literature search on PubMed, Web of Sciences, and Scopus was performed to find studies on HRQoL in transplant β-TM patients. The studies were published from January 1st, 2000 to September 30th, 2020. Using MeSH headings, we searched for the terms “bone marrow transplantation”, “stem cells transplantation”, “allogeneic”, “quality of life”, “HRQoL”, “Health-related quality of life,” “thalassemia”,” β-thalassemia”, “thalassemia major” as well as variations thereof. A more extensive description of the search strategy is shown in Table **S1**. The results were defined using the Preferred Reporting Items for Systematic and Meta-analyses (PRISMA) statement to identify, select, and determine the eligibility of papers for inclusion in the study. The PRISMA 2009 checklist is attached in (Fig. **1**) shows the study flow diagram. Quality rating of studies was performed by NIH Study Quality Assessment Tools through different questions that evaluate several domains [10] and the results are shown in Table **S2**.

### 2.2 Inclusion and Exclusion Criteria

According to the standard PICOS approach, we defined the following eligibility criteria: our participants (P) are thalassemia patients, the intervention (I) is hematopoietic stem cell transplantation, the comparison (C) group includes non-transplanted thalassemia patients and the general population, and the primary outcome (O) is the quality of life. Studies were included in this study if: (1) they were published in peer-reviewed journals; (2) they were published in the English language; (3) they reported evaluation of HRQoL in β-TM patients that underwent HSCT in comparison with β-TM patients treated with conventional therapy, other transplantable diseases, or the general population; (4) they were longitudinal or cross-section studies.

Studies were excluded if: (1) they were in languages other than English; (2) they were case series, reviews, letters, editorials, or commentaries; (3) they reported an analysis of HRQoL of β-TM patients without distinctions between treatments. Two reviewers (O.M. and I.P.) autonomously identified studies for potential inclusion. After exclusion of duplicates (including articles repeatedly reporting the results of the same trial or with overlapping samples) and articles that were unrelated to the main topic, individual studies were included when they matched the inclusion criteria. Discrepancies between the reviewers were resolved by consulting a third experienced researcher (G.LN.).

### 2.3 Statistical Analysis

In one study, data of mean and standard deviation was not available [11] and was calculated from median and ranges according to Luo *et al*. and Wan *et al*. [12, 13]. Statistical analyses were performed through the t-test on dependent variables. Standard mean difference and a 95% confidence interval (CI) were calculated. To assess heterogeneity between the studies, the chi-squared test (for evaluation of heterogeneity between studies statistically; P less than 0.05) and I^2^ index (to evaluate the heterogeneity of the results) were used. All meta-analyses were conducted using Review Manager 5.4 and were performed by pooling the standard effect sizes using a random-effects model. This model assumes that within-group variability in scores and mean effect size are caused by differences between studies. The common metric in the study was the standardized mean difference according to Cohen (Cohen’s *d*), which is an appropriate effect size comparison between two means. Cohen’s *d* was used to estimate effect size using the following interpretation: small (0.2), medium (0.5), and large (0.8).

The QoL questionnaires utilized were QLQ-C30 [14], the Medical Outcomes Study 36-Item Short-Form Health Survey (SF-36) [15], WHOQOLBREF (HK) [16, 17], and PedsQL questionnaires [18, 19]. From each measure, several dimensions were considered: physical, emotional, and social function and a more accurate description of each questionnaire can be found in the Table **S3**. If available, the Pesaro risk classification was evaluated. This classification predicts outcomes of hematopoietic stem cell transplantation for TM patients. Three variables were found to significantly influence transplant outcomes: hepatomegaly (defined in terms of centimetres below the costal arch), liver fibrosis (absent or present), and chelation history (regular or irregular) [20].

## 3 RESULTS

We initially screened 247 articles; after the removal of duplicates, we recorded 218 articles and kept 33; 10 of the 33 articles were assessed for eligibility (Fig. **1**). Twenty-three articles were excluded. Of these, 12 did not evaluate QoL outcomes or bone marrow transplant patients; 2 did not consider β-TM patients or lacked in principal outcomes;7 were reviews, and 2 articles did not clearly distinguish between β-TM patients who underwent transplantation. Finally, 10 studies were included in the qualitative synthesis and 5 in the quantitative. Based on the inclusion and exclusion criteria, 10 articles were included [11, 21-29] for qualitative analysis, with a total sample of 395 patients examined. The total number of patients included in the quantitative analysis was 576. Transplanted β-TM patients were compared with β-TM patients treated with standard therapy such as red cells transfusion and iron chelation therapy. A small number of studies have compared transplanted β-TM patients to the general population or transplanted patients with other hematologic diseases.

Overall, 5 papers were considered for the quantitative analysis between transplanted and conventionally treated β-TM patients [11, 21, 24, 25, 28]. Full characteristics of the studies examined in this systematic review are resumed in Table **1**.

### 3.1 Qualitative Analysis

Javanbakht *et al*. compared 44 transplanted β-TM patients with 74 transfusion-dependent patients in Iran. The time elapsed between transplant and HRQoL assessment was 8 months and 18 years. Physical and emotional domain scores, evaluated by using SF-36, were statistically higher in transplant patients (physical function: 93.07 *vs*. 84.93, P=0.004; role limitations due to physical problems: 77.84 *vs*. 58.88, P=0.003; role limitations due to emotional problems: 78.79 *vs*. 55.92, P=0.001). The authors concluded that HSCT give better HRQoL compared to standard therapy [21].

Caocci *et al*. investigated HRQoL in a cross-sectional study of 19 β-TM patients, evaluated by EORTC QLQ-C30. The time elapsed between transplant and HRQoL assessment was 300 days or more. The mean of general HRQoL after unrelated donor HSCT was good (mean score: 76.4). The comparison of patients with or without GVHD showed that the mean of general HRQoL was good (mean score: 65.3) in the first cohort and very good (mean: 81.9) in the second one. No statistical difference was found in global health analysis but patients without GVHD reported better value in physical function (95 *vs*. 82.2, P=0.04), emotional function (89.6 *vs*. 66.7, P=0.01), pain (4.2 *vs*. 33.3, P=0.01), and insomnia (0 *vs*. 27.8, P=0.02) [22].

La Nasa *et al*. compared 109 β-TM transplant patients with the general population and with another control cohort of 124 β-TM patients treated conventionally, evaluated by SF-36 and FACT-BMT. The median time elapsed between transplant and HRQoL assessment was 22.8 years (range: 11.7-30.3). The comparison of β-TM HSCT patients and the general population showed lower scores in the general health scale (69.9 *vs*. 72.2, P=0.005). However, the sub-analysis on GVHD showed higher scores in β-TM transplanted patients without GVHD compared with the general population in mental health (11.1; 95%CI, 3.8-18.3, P=0.003), role emotional functioning (15.9; 95% CI, 4.5-27.3; P=.007), and mental component summary (5.3; 95% CI, 1.6-9, P=0.006). Worse scores were observed in the patients >15-years for general health (-12.8, 95%CI, -23.1 to -2.6, P=0.015) and physical component summary (-4.3, 95%CI, -8.2 to -0.3, P=0.035). In addition, better outcomes were found in transplanted patients compared with transfusion-dependent patients, especially in role limitations due to physical functioning (94.50 *vs*. 71.45), bodily pain (84.53 *vs*. 63.29), and role emotional functioning (92.05 *vs*. 74.37) scales [24].

Caocci *et al*. collected HRQoL data, by SF-36, on some patients evaluated in the previous paper and compared 71 β-TM transplant patients with their sibling donors and another group of 71 conventionally treated patients. The median time elapsed between transplant and HRQoL assessment was 21 years. Physical health scores of transplanted patients were not statistically different from those of their sibling donors (68.4 *vs*. 70.4, P = NS) but were significantly higher in comparison with patients under transfusions and iron chelation (68.4 *vs*. 61.6, P = 0.01) [29].

Cheuk *et al*. evaluated a transplant β-TM group of 24 patients and a conventional treatment group of 74 patients by different questionnaires. The median time elapsed between transplant and HRQoL assessment was 6.5 years (range: 1.1-13.5 years). In the cohort of patients older than 12 years, the WHOQOL-BREF(HK) questionnaire was administered. Overall health and physical health were better in the transplant group in comparison with the transfusion group (3.67 *vs*. 3.06, P=0.01 and 75.20 *vs*. 63.94, P=0.007, respectively). Similarly, the personal relationship domain was better in transplanted patients (4.13 *vs*. 3.69, P=0.014). Subdivision by the Pesaro score, which evaluates the mortality risk of transplantation in β-TM patients based on hepatomegaly, liver fibrosis, and compliance to iron chelation [20], showed that the class II of risk has a significantly higher score in the psychological domain than class III (72.6 *vs*. 56.2, P=0.042). No differences were found in psychological, social relationships, and environment domains. In the other cohort (<18 years), the PedsQL questionnaire was used. The overall scores in emotional, social, psychosocial, and school domains were not significantly different between the transplant and non-transplant groups. Scores in the physical domain were similar in the 2 groups, but transplant patients reported better scores in running (3.53 *vs*. 2.72, P=0.001) and sports exercises (3.2 *vs*. 2.64, P=0.038) [28].

Uygun *et al*. split patients by age and showed that higher HRQoL scores were found in the group of 49 patients aged between 5-18 years and those who underwent HSCT, compared to the group of 50 non-transplanted age-matched (77.65 *vs*. 71.77 P=0.045) patients. Particularly, in 5-7-year-old patients, differences were found in the emotional domain (77.5 *vs*. 54 P=0.035), while in the 8-12-year-old group, differences were present in the physical and school domains (81.3 *vs*. 69 P=0.012 and 79.5 *vs*. 70.7 P=0.046). In the adult group, the physical domain and the overall health showed higher scores in transplanted patients compared to the transfusion group (79.7 *vs*. 66.6, P=0.041 and 80.5 *vs*. 60.4 P=0.034, respectively). The presence of GVHD decreased HRQoL in transplanted patients (81.5 *vs*. 65.5 P=0.030). The median time elapsed between transplant and HRQoL assessment was 4.4 years (range: 2.01-11.96 years). HRQoL was assessed usingWHOQOL-BREF (HK) and PedsQL instruments. Due to these results, the authors concluded that HSCT should be performed before primary school [26].

Patel *et al*., in a recent paper, showed similar results using PedsQL in two Indian β-TM cohorts. The median time elapsed between transplant and HRQoL assessment was 5 years (range: 2-10 years). In the transfusion group (60 patients), HRQoL scores were lower than in the transplanted cohort (40 patients), especially in physical (83.6 *vs*. 92.8, P <0.001), emotional (84.4 *vs*. 93.5, P <0.001), school (74.8 *vs*. 88.6, P <0.001), and psychosocial domains (82.7 *vs*. 91.6, <0.001). Significant differences among means of same domains were found when the comparison was made between transplanted children and transfusion-dependent adult patients in physical (73.5 *vs*. 92.8, P <0.001), emotional (74.5 *vs*. 93.5, P <0.001), school (67 *vs*. 88.6, P <0.001), and psychosocial domains (76 *vs*. 91.6, <0.001) [25].

Bahar *et al*. found better HRQoL scores in 84 β-TM patients who underwent HSCT compared to 71 non-transplanted patients in psychological (65.1 *vs* 75.2), social relationship (24.3 *vs* 24.1), and physical domains (116.7 *vs* 107.2) [27]. The measurement of HRQoL was assessed in patients using the KINDL scale 6 months after the transplant.

Caocci *et al*. reported HRQoL longitudinal data in a study where 28 β-TM Middle Eastern patients were evaluated through PedsQL questionnaire, before transplant and at different time points after this procedure, particularly after 3, 6, and 18 months post-transplant. The transplant impacted the general well-being with a reduction of mean scores from baseline to 3 months after HSCT (81 *vs*. 75.7). A progressive increase was observed at 6 months post-HSCT with 80.6 points and at 18 months post-HSCT with 94.4 points. The difference between baseline and the last time-point was statistically significant (P=0.02). A similar trend was observed in the physical functioning domain [11].

Finally, Kelly *et al*. compared 13 β-TM patients and a group of 268 patients with hematological acquired diseases and analyzed their HRQoL differences before and after HSCT. The patients were evaluated using CHRIS-General domains before transplant and 45 days as well as 3, 6, and 12 months after transplant. Children with hemoglobinopathies had higher physical and emotional functioning scores prior to HSCT but they experienced a similar pattern of recovery to their baseline functioning after three months post-HSCT when compared to children receiving HSCT for acquired conditions [23].

Overall, the quality of studies collected was good except for one that had low scores (Table **S2**).

### 3.2 Quantitative Analysis

Only the comparison between HSCT and transfusion-dependent β-TM patients was considered for quantitative analysis. The analysis was based on 3 HRQoL principal domains: physical, emotional, and social function, and a total of 5 articles were included with 439 patients collected [12, 15-17, 20]. The forest plots with the results are shown in Fig. (**2**). The random-effect analysis of physical function revealed a significant difference between transplant β-TM and transfusion-dependent β-TM patients, with a medium effect size [*d*=0.65, 95% CI (0.29-1.02), z = 3.52, p =0.0004]. For emotional function, the effect size was medium again, with significant difference [*d*=0.59, 95% CI (0.43-0.76), z = 6.99, p <0.00001]. Significant results were found in the social function domain but with a small effect size between the groups [*d*=0.38, 95% CI (0.02-0.74), z = 2.05, p =0.04]. There was evidence of substantial heterogeneity in the physical and social function domains (I^2^=75% in both domains) but not in the emotional domain (I^2^=0%). The risk of bias was evaluated in all of the studies and scores were good except for one [25].

## 4 DISCUSSION

β-TM can currently benefit from a valid support therapy (transfusion and iron chelation) that, if well conducted, offers a life expectancy similar to that of the healthy population. HSCT is the only treatment capable of assuring definitive healing. Unfortunately, the choice of a transplant procedure in a non-malignant chronic disease is not easy, because of the risk of transplant-related mortality and the clinical complications following HSCT [30]. Ensuring a better HRQoL after HSCT could therefore represent one of the elements that need to be taken into account for the clinical decision-making process. However, few studies have assessed HRQoL in β-TM patients’ literature, and the samples examined frequently differ in age, type of comparison, and questionnaire used. (Fig. **1**) describes the article selection process.

HRQoL of transplanted β-TM patients was not different when the comparison was made with other patients transplanted for other conditions, i.e., non-hemoglobinopathies [23]. As expected, in comparison to the general population, lower scores were found in the general health scale [24], but not in the physical health scores [29]. After the transplant procedure, there was a significant increase in HRQoL profile, especially in well-being and physical function [11].

Age at the time of transplantation has a crucial influence on HRQoL. In general, β-TM patients show more anxiety and depression as they age [21]. A comparison of HSCT β-TM children and transfusion-dependent patients revealed lower scores in different domains in the conventionally treated cohort with the worst results found when comparing adults β-TM transfusion-dependent patients [25]. An analysis made in patients aged between 5 and 18 years showed a higher average HRQoL in the transplant group compared with standard support therapy. Sub analysis confirmed differences in the emotional domain in 5 to 7-year-old patients and in the physician and school domains in the 8-12-year-old group [26]. Even distinction between groups of transplanted patients aged more or less than 15 years was evaluated. Worse scores were observed in the patients >15-years for general health and physical component summary [24]. Conversely, other authors comparing patients aged less than 18-years (median age 11.1 years, 5.3-17.9) and patients aged more than 12-years (median age 18.2 years, 12.3-30.4) found significant differences in the second group. In particular, they found differences in overall health and overall physical health between transplant and transfusion groups. Similarly, personal relationship score was higher in transplanted patients. In the group of only pediatric patients, better scores were reported in the running domain and sports exercises but they had lower scores in school absence due to frequent access to the hospital [28] because they needed to attend regular follow-up in the post-transplant period.

Besides age, another important factor influencing a good quality of life is GVHD. GVHD decreases HRQoL even after 2 years since transplantation in children and a similar reduction was reported in parents’ score [26]. Differences between the groups with or without GVHD were also present in the physical function, emotional function, pain, and insomnia [22]. Interestingly, HSCT β-TM patients without GVHD in mental health, role emotional functioning, and mental component summary had higher scores compared with the general population [24], suggesting that HSCT is considered as a possible new beginning in these patients.

**Fig. (2):**
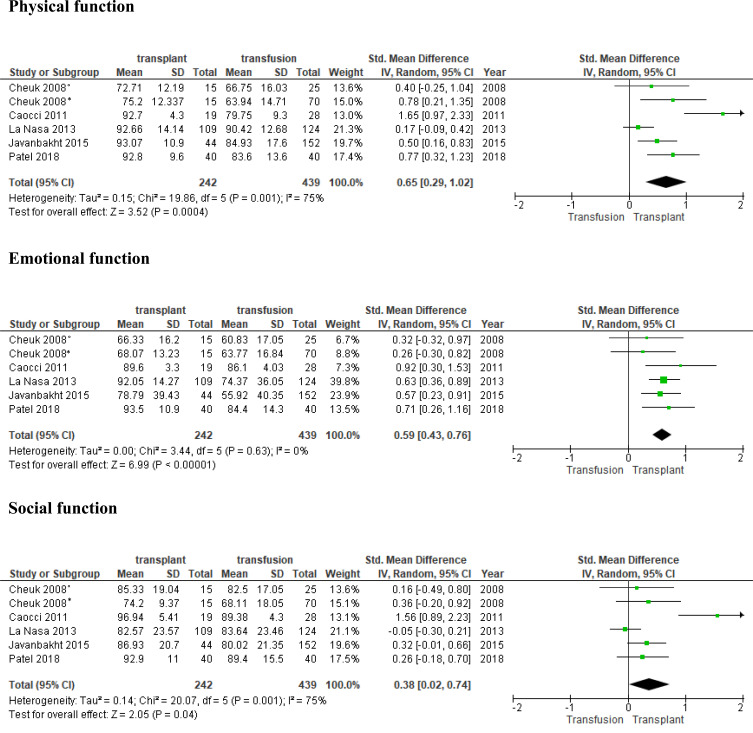
°patients aged < 18 years, * patients aged ≥12 years.

A more complete vision, however, is given by the analysis between HSCT and transfusion dependent β-TM patients. Overall, analysed HRQoL domains were better in transplanted patients (Fig. **2**). In particular, higher scores were found in overall health [26, 28]. Interestingly, all studies recorded higher significant scores in physical domains, ranging between 68.4 and 116.7 [21, 24-29]. The effect size of 5 articles considered in the meta-analysis confirm this trend. Social relationship and psychosocial domains also showed better results [25, 27, 28]. The social function domain showed a significant result but caution in interpretation is necessary because of the small effect size and high heterogeneity. The most interesting results are those related to the emotional function. Indeed, 4 papers reported better significant scores in transplanted patients [10, 13, 15, 16]. Data are corroborated by pooled analysis with medium effect size with significant difference and weak heterogeneity. A recent meta-analysis comparing HRQoL between transfusion dependent β-TM and general population found that patients transfusion dependent β-TM had lower physical and mental health scores [31]. This confirms that the pathology negatively affects different aspects of life. The transplant could fill these gaps and improve the patient’s quality of life.

The current analysis has several limitations. First, only few papers are available for this analysis. The power of results was thus limited. There was heterogeneity in questionnaires utilized. Because some data were missing, meta-regression was not applicable in exploring the related factors such as sex, age, ferritin values. However, the analysis of the available data has emphasized the positive role of transplantation in this category of patients, which has been shown to improve HRQoL.

## CONCLUSION

The results of the present study suggested that HSCT in β-TM is associated with positive outcomes in different domains of HRQoL. Novel therapeutic strategies arise in the panorama of β-TM treatments [32, 33]. In this contest, HRQoL appears to be a good instrument for ensuring a better treatment decision. Prospective studies should be conducted to confirm the results obtained.

## Figures and Tables

**Table 1:** Characteristic of principals studies collected.

**Author**	**Year**	**N° Patients**	**Mean Age Years, (Range) of Transplanted Patients**	**Pesaro Stade TM**	**Type Questionnaire**	**Study Design**	**Comparison Cohort**
Bahar [27]	2005	84	9.5	ND	KINDL	Cross-sectional	No transplant beta-thalassemia
Caocci [22]	2006	19	22 (17-37)	III	EORTC QLQ-C30	Cross-sectional	None
Cheuk [28]	2008	24	15.2 (5.3-30.4)	I, II, III	WHOQOL-BREF(HK), PedsQL	Cross-sectional	No transplant beta-thalassemia
Caocci [11]	2011	28	10 (5-17)	II, III	PedsQL	Longitudinal	Same patients before transplant
Kelly [23]	2012	6	8 (5-18)	ND	CHRIS-General domains	Longitudinal	Transplant for other hematological diseases
Uygun [26]	2012	49	11.6 (3.5-27.5)	ND	WHOQOL-BREF Peds QoL	Cross-sectional	No transplant beta-thalassemia
La Nasa [24]	2013	109	34 (21-48)	I, II, III	SF-36, FACT-BMT	Cross-sectional	No transplant beta-thalassemia and health
Javanbakht [21]	*2015*	44	20 (5-35)	ND	SF-36	Cross-sectional	No transplant beta-thalassemia
Caocci [29]	2016	71	33	ND	SF-36	Cross-sectional	No transplant beta-thalassemia and health
Patel [25]	2018	40	10 (5-18)	ND	Peds QoL	Cross-sectional	No transplant beta-thalassemia
