# Supplementary material for: Systematic Review and Meta-Analysis of Health-Related Quality of Life in Patients with β-Thalassemia that Underwent Hematopoietic Stem Cell Transplantation
Source: Clin Pract Epidemiol Ment Health. 2023 Dec 10;19(Suppl-1):e174501792301031. doi: 10.2174/17450179-v17-e211208-2021-HT2-1910-4 (PMC11037551; doi:10.2174/17450179-v17-e211208-2021-HT2-1910-4)
Supplement: Supplementary file 1 [file CPEMH-19-e174501792301031_SD1.pdf]

1745-0179/23 *Send Orders for Reprints to reprints@benthamscience.net*

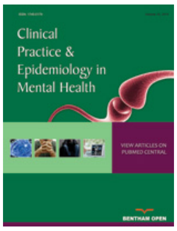

Clinical Practice & Epidemiology in Mental Health

Content list available at: https://clinical-practice-and-epidemiology-in-mental-health.com

Supplementary Material

Systematic Review and Meta-Analysis of Health-Related Quality of Life in Patients with β-Thalassemia that Underwent Hematopoietic Stem Cell Transplantation

Olga Mulas[1,*], Ilaria Pili[1], Marco Sanna[1] and Giorgio La Nasa[1]

[1]*Ematologia e CTMO, Ospedale Businco, ARNAS "G. Brotzu", Cagliari, Italy*

Table S1. Search strategy

Database	Search Strategy
pubmed	("beta thalassaemia"[All Fields] OR "beta thalassemia"[MeSH Terms] OR "beta thalassemia"[All Fields] OR ("beta"[All Fields] AND "thalassemia"[All Fields]) OR "beta thalassemia"[All Fields]) AND ("hrqols"[All Fields] OR "quality of life"[MeSH Terms] OR ("quality"[All Fields] AND "life"[All Fields]) OR "quality of life"[All Fields] OR "hrqol"[All Fields]) AND ("hematopoietic stem cell transplantation"[MeSH Terms] OR ("hematopoietic"[All Fields] AND "stem"[All Fields] AND "cell"[All Fields] AND "transplantation"[All Fields]) OR "hematopoietic stem cell transplantation"[All Fields] OR "hsct"[All Fields])

Table S2. Quality Assessment Tool for Observational Cohort and Cross-Sectional Studies; NA: not applicable, Q: question

Author	Year	Q1	Q2	Q3	Q4	Q5	Q6	Q7	Q8	Q9	Q10	Q11	Q12	Q13	Q14	Total
Bahar [27]	2005	Yes	Yes	NA	Yes	NA	No	NA	NA	Yes	No	Yes	NA	NA	No	5
Caocci [22]	2006	Yes	Yes	NA	Yes	NA	No	NA	NA	Yes	No	Yes	NA	NA	Yes	6
Cheuk [28]	2008	Yes	Yes	NA	Yes	NA	No	NA	NA	Yes	No	Yes	NA	NA	Yes	6
Caocci [11]	2011	Yes	Yes	NA	Yes	NA	Yes	NA	NA	Yes	Yes	Yes	NA	No	Yes	8
Kelly [23]	2012	Yes	Yes	NA	No	NA	Yes	NA	NA	Yes	Yes	Yes	NA	Yes	Yes	8
Uygun [26]	2012	Yes	Yes	NA	Yes	NA	No	NA	NA	Yes	No	Yes	NA	NA	Yes	6
La Nasa [24]	2013	Yes	Yes	NA	Yes	NA	No	NA	NA	Yes	No	Yes	NA	NA	Yes	6
Javanbakht [21]	2015	Yes	Yes	NA	Yes	NA	No	NA	NA	Yes	No	Yes	NA	NA	Yes	6
Caocci [29]	2016	Yes	Yes	NA	Yes	NA	No	NA	NA	Yes	No	Yes	NA	NA	Yes	6
Patel [25]	2018	Yes	No	NA	Yes	NA	No	NA	NA	Yes	No	Yes	NA	NA	No	4

Legend of items analysed

Q1: Was the research question or objective in this paper clearly stated?
Q2. Was the study population clearly specified and defined?
Q3. Was the participation rate of eligible persons at least 50%?
Q 4. Were all the subjects selected or recruited from the same or similar populations (including the same time period)? Were inclusion and exclusion criteria for being in the study prespecified and applied uniformly to all participants?
Q 5. Was a sample size justification, power description, or variance and effect estimates provided?
Q 6. For the analyses in this paper, were the exposure(s) of interest measured prior to the outcome(s) being measured?
Q 7. Was the timeframe sufficient so that one could reasonably expect to see an association between exposure and outcome if it existed?
Q 8. For exposures that can vary in amount or level, did the study examine different levels of the exposure as related to the outcome (*e.g.*, categories of exposure, or exposure measured as continuous variable)?
Q 9. Were the exposure measures (independent variables) clearly defined, valid, reliable, and implemented consistently across all study participants?
Q 10. Was the exposure(s) assessed more than once over time?
Q 11. Were the outcome measures (dependent variables) clearly defined, valid, reliable, and implemented consistently across all study participants? Q 12. Were the outcome assessors blinded to the exposure status of participants?
Q 13. Was loss to follow-up after baseline 20% or less?
Q 14. Were key potential confounding variables measured and adjusted statistically for their impact on the relationship between exposure(s) and outcome(s)?

DOI: 10.2174/17450179-v17-e211208-2021-HT2-1910-4, 2023, *19*, (Suppl-1, M3) e174501792301031-SP

Table S3. Brief description of questionnaires utilized in the articles.

Questionnaires	Description
EORTC QLQ-C30	Created by the European Organization for Research and Treatment of Cancer (EORTC) QoL study group, this questionnaire was developed to assess the global quality of life of oncology patients, through measurement of physical, psychological, and social function.
SF-36	This questionnaire is a survey on the patient's global health status, assessed by physical component summary and mental component summary (MCS) scores, which represents the aggregation of eight domains on the 36-item Short-Form Health Survey
WHOQOLBREF (HK)	This questionnaire comprises 24 global questions and 2 national questions and it assesses a patient’s QoL in four domains: physical health, psychological, social relationships and environment.
PedsQL questionnaires	Pediatric Quality of Life Inventory. This 23-item multidimensional questionnaire was designed to evaluate the essential score domains for pediatric HRQoL, including physical, emotional, and social functioning as defined by the World Health Organization, as well as school functioning
FACT-BMT	Functional Assessment of Cancer Therapy–Bone Marrow Transplant (FACT-BMT) scale, a self-administered tool used to assess multidimensional domains of HRQoL in patients after HSCT. It consists of the 27-item FACT-General and the 23-item Bone Marrow Transplantation Subscale. The FACT-G measures 4 dimensions of QoL physical well-being, social/family well-being, emotional well-being, and functional well-being.
KINDL	This questionnaire is developed for assessing HRQoL in children and adolescents aged 3 years and older and it comprises 24 items, with several domains
CHRIS-General domains	This questionnaire contains 20 items forming three general health status domains: physical, role, and emotional functioning. The measure is designed to assess the child’s status in the week prior to the assessment using separate parent and child versions. The measure also contains a summary item of the child’s general health (parent report, only).

© 2023 Mulas *et al*.

This is an open access article distributed under the terms of the Creative Commons Attribution 4.0 International Public License (CC-BY 4.0), a copy of which is available at: (https://creativecommons.org/licenses/by/4.0/legalcode). This license permits unrestricted use, distribution, and reproduction in any medium, provided the original author and source are credited.
